# Nampt/PBEF/Visfatin Upregulation in Colorectal Tumors, Mirrored in Normal Tissue and Whole Blood of Colorectal Cancer Patients, Is Associated with Metastasis, Hypoxia, IL1*β*, and Anemia

**DOI:** 10.1155/2015/523930

**Published:** 2015-05-13

**Authors:** Katarzyna Neubauer, Iwona Bednarz Misa, Dorota Diakowska, Bartosz Kapturkiewicz, Andrzej Gamian, Malgorzata Krzystek-Korpacka

**Affiliations:** ^1^Department of Gastroenterology and Hepatology, Wroclaw Medical University, Borowska 213, 50-556 Wroclaw, Poland; ^2^Department of Medical Biochemistry, Wroclaw Medical University, Chalubinskiego 10, 50-368 Wroclaw, Poland; ^3^Department of Gastrointestinal and General Surgery, Wroclaw Medical University, M. Curie-Sklodowskiej 66, 50-369 Wroclaw, Poland; ^4^First Department of Oncological Surgery, Lower Silesian Oncology Center, Plac Hirszfelda 12, 53-413 Wroclaw, Poland; ^5^Wroclaw Research Center EIT+, Stablowicka 147, 54-066 Wroclaw, Poland

## Abstract

Targeting Nampt/PBEF/visfatin is considered a promising anticancer strategy, yet little is known about its association with colorectal cancer (CRC). We quantified Nampt/PBEF/visfatin expression in bowel and blood (mRNA and protein), referring it to CRC advancement and inflammatory, angiogenic, hypoxia, and proliferation indices. Tumor Nampt/PBEF/visfatin upregulation was associated with metastasis, anemia, tumor location, *HIF1α*, and inflammatory and angiogenic indices, of which *HIF1α*, *IL1β*, and anemia explained 70% in Nampt/PBEF/visfatin variability. Nampt/PBEF/visfatin expression in nontumor tissue, both mRNA and protein, increased in patients with metastatic disease and mild anemia, and, on transcriptional level, correlated with *HIF1α*, *IL1β*, *IL8*, *CCL2*, and *CCL4* expression. Whole blood Nampt/PBEF/visfatin tended to be elevated in patients with metastatic cancer or anemia and correlated with inflammatory indices, of which *IL1β*, *IL8*, and hematocrit explained 60% of its variability. Circulating visfatin was associated with lymph node metastasis and inflammatory and angiogenic indices. *In vitro* experiments on SW620 cells demonstrated Nampt/PBEF/visfatin downregulation in response to serum withdrawal but its upregulation in response to serum induction and hypoxia. Stimulation with recombinant visfatin did not provide growth advantage. Summarizing, our results link Nampt/PBEF/visfatin with tumor metastatic potential and point at inflammation and hypoxia as key inducers of its upregulation in CRC.

## 1. Introduction

Nicotinamide phosphoribosyltransferase (Nampt)/pre-B-cell colony enhancing factor (PBEF)/visfatin is gaining attention as a target of novel anticancer therapies. However, its exact role in physiology and disease and the mechanisms underlying Nampt/PBEF/visfatin actions remain to be clarified. Up to date, the best established one is that of an intracellular enzyme (iNampt) in bioenergetics, cellular metabolism, and DNA repair. As a rate-limiting enzyme in the salvage pathway of NAD biosynthesis from nicotinamide, iNampt is a key regulator of NAD utilizing enzymes, namely, dehydrogenases, sirtuins, poly(ADP-ribose) polymerases, and mono-ADP-ribosyl transferases. In turn, its extracellular form (eNampt) is a cytokine/growth factor displaying hematopoietic, immunomodulating, proinflammatory, angiogenic, chemotactic, and antiapoptotic properties. As a visfatin secreted mainly by visceral fat, it has been claimed to function as an insulin-mimetic hormone [1–3]. Despite the controversies around Nampt/PBEF/visfatin, its key inhibitors, APO866 and GMX1778, have already entered clinical trials as anticancer agents [2, 3]. However, considering diversity of biological processes in which Nampt/PBEF/visfatin might be implicated, discerning its exact role in pathomechanisms of a variety of diseases prior to devising Nampt/PBEF/visfatin-targeting strategies ought to be priority if serious side-effects are to be avoided. The need is emphasized by the recent discovery of Pittelli et al. [4] showing that Nampt/PBEF/visfatin activity is necessary not only for activated but also for resting lymphocytes and its inhibition results in their apoptosis.

Both circulating levels and tissue expression of Nampt/PBEF/visfatin have been demonstrated to be upregulated in cancer, in which Nampt/PBEF/visfatin is believed to contribute to the disease progression by inducing proliferation, survival, and angiogenesis [2]. Although colorectal cancer (CRC) remains one of the commonest cancers worldwide, data on Nampt/PBEF/visfatin expression in CRC are surprisingly scanty. Comparing tissue from rectal adenocarcinoma (stage III) with adjacent nonneoplastic mucosa from a single patient, Hufton et al. [5] were first to demonstrate Nampt/PBEF/visfatin overexpression in cancer. Nampt/PBEF/visfatin upregulation in CRC has been further confirmed both on transcriptional and protein level in 6 out of 8 investigated samples [6] and the possible relevance for cancer progression has been hinted but not explored. Circulating visfatin has been investigated as well and its association with CRC advancement has been initially reported [7] but not confirmed [8, 9]. Although activated leukocytes are well known sources of Nampt/PBEF/visfatin [10], its status in leukocytes of CRC patients is unknown. Hence, the aim of this study was to evaluate tissue and whole blood expression of Nampt/PBEF/visfatin in reference to the disease advancement and to identify factors that might be involved in its upregulation in order to gain insights into the potential role of Nampt/PBEF/visfatin in CRC.

## 2. Materials and Methods

### 2.1. Patients

For Nampt/PBEF/visfatin transcriptional analysis, 51 CRC patients admitted to Gastrointestinal and General Surgery Department of Wroclaw Medical University or Lower Silesian Oncology Center for Curative Resection were enrolled. Pairs of bowel tissue, derived from adenocarcinomas and resection margin (histopathologically confirmed to be tumor-free), were collected, soaked in RNAlater (Ambion, USA), and stored at −80°C.

Whole blood samples (3 mL) were collected prior to any treatment from 36 CRC patients into PAXgene Blood RNA Tubes. 54 whole blood samples from patients with polyps, nonactive inflammatory bowel disease, or irritable bowel syndrome from of Gastroenterology and Hepatology Department served as noncancer controls.

Characteristics of study population are given in Tables 1 and 2. There was significant (*p* < 0.001) difference in age distribution between CRC patients and controls (Table 2). However, Nampt/PBEF/visfatin did not correlate with age either in a whole cohort (*r* = 0, *p* = 0.988) or in CRC (*r* = 0, *p* = 0.992) or control (*r* = 0.20, *p* = 0.154) groups.

For Nampt/PBEF/visfatin analysis on protein level, matched serum and tissue samples from 26 CRC patients were examined. Bowel tissue samples collected for protein analysis (matched cancerous and noncancerous) were rinsed with 0.9% NaCl and deep-frozen while these for comparative mRNA analysis were rinsed and stored in RNAlater. Additionally, serum samples from apparently healthy blood donors from Lower Silesian Center of Blood Donation and Therapy, Wroclaw, Poland (recruited on the basis of standard eligibility criteria for blood donation with an age >40 yrs old as an inclusion criterion for the current study), as well as otherwise healthy patients submitted for hernia repair surgery or surgical removal of hemorrhoids were obtained (*n* = 48). Age of patients was 69.2 ± 12.1 as compared to 65.9 ± 12.5 in controls, *p* = 0.281, and female to male ratio was, respectively, 11/15 and 26/22, *p* = 0.466. Within this group of patients, there were four with stage I, 12 with stage II, seven with stage III, and three with stage IV CRC; eight patients with T2 cancers, 13 with T3, and five with T4; and 18 patients without and eight with lymph node involvement.

Mild anemia was defined as hemoglobin level <13 g/dL in man and <12 g/dL in woman; moderate anemia was defined as hemoglobin <9.5 g/dL and severe anemia as hemoglobin <8 g/dL.

### 2.2. Ethical Approval

The study protocol was approved by the Medical Ethics Committee of Wroclaw Medical University, Wroclaw, Poland, and the study was conducted in accordance with the Helsinki Declaration of 1975, as revised in 1983, and an informed consent has been obtained from patients.

### 2.3. Analytical Methods

For Nampt/PBEF/visfatin analysis on mRNA level, tissue samples (30–40 mg) were homogenized using Fastprep 24 Homogenizer (MP Biomedical, USA). RNA was isolated using RNeasy Plus Mini Kit (Qiagen, USA) with DNase treatment. Whole blood RNA was isolated with PAXgene Blood RNA Kit (Qiagen). Isolated RNA was quantified with NanoDrop 2000 (ThermoScientific, USA). Its purity was assessed by calculating 260/280 and 260/230 ratios. RNA integrity was evaluated using the Experion platform incorporating LabChip microfluidic technology and Experion RNA StdSens analysis kits (BioRad, USA). Only RNA isolates with quality indicator (RQI) ≥ 7 were used (1, degraded; 10, intact). Contamination with inhibitors was tested by calculating PCR efficiencies (five-fold dilutions, 6-point curve, in duplicate). 0.5 *μ*g of tissue or 0.25 *μ*g of whole blood RNA/20 *μ*L was transcribed using Maxima First Strand cDNA Synthesis Kit for RT-qPCR (Thermo Scientific). Negative transcription controls were prepared and tested for all samples. qPCRs were conducted in triplicate using CFX96 Real-Time PCR system (BioRad) and SsoFast EvaGreen Supermix (BioRad) with cycling conditions: 30 sec activation at 95°C, 5 sec denaturation at 95°C, and annealing/extension for 5 sec at 61°C, 40 cycles, followed by melting step (60–95°C with fluorescent reading every 0.5°C). Reaction mixture contained 2 *μ*L of cDNA (diluted 1 : 5), 10 *μ*L of 2x SsoFast EvaGreen Supermix, 1 *μ*L of each 10 nM forward and reverse target-specific primers, and 6 *μ*L of water.

*SDHA* and* TBP* have previously been selected as optimal normalizers for CRC whole blood samples (manuscript submitted) and* GAPDH* and* PPIA* for colorectal tissue [11]. Primers (Generi Biotech, Czech Republic), spanning at least one intron, were devised, optimized and validated as previously described [11]. Their sequences and efficiencies are presented in Table 3.

For Nampt/PBEF/visfatin analysis on protein level, tissue samples (120–170 mg) were homogenized 1 : 2 (w/v) in 10 mM Tris-HCl with 1 mM EDTA, pH 7.4 buffer, in FastPrep-24 homogenizer (2 min., 4.0 M/S). Homogenates were centrifuged (14 500 ×g, 10 min., 6°C) and the supernatants were aliquoted and stored at −45°C until analysis.

For circulating Nampt/PBEF/visfatin analysis, blood was drawn by venipuncture and allowed to clot for 30 minutes and centrifuged (15 min., 720 ×g) and serum was collected, aliquoted, and kept frozen at −80° until examination.

Nampt/PBEF/visfatin in sera and tissue homogenates was measured by means of flow cytometry-based method utilizing magnetic microspheres conjugated with monoclonal antibodies using the BioPlex 200 platform with HRF (Bio-Rad, USA), incorporating Luminex xMAP technology, and Bio-Plex Pro Human Diabetes Assay Panels. In case of tissue Nampt/PBEF/visfatin, its concentrations were adjusted to total protein level, measured using Bradford method with bovine serum albumin used for standard curve preparation, and expressed as mg of Nampt/PBEF/visfatin per g of total protein content.

Biochemical and hematological parameters were retrieved from medical records. Data on circulating levels of cytokines/growth factors, assessed using Luminex xMAP technology, were retrieved from earlier study [12].

#### 2.3.1. Cell Cultures

Human colon adenocarcinoma cell line SW620 (ATCC: CCL-227) from the Polish Collection of Microorganisms (PCM) of the Institute of Immunology and Experimental Therapy of Polish Academy of Science, Wroclaw, Poland (PCM-TC046), was grown on 75 cm^2^ cell culture flasks (BD Bioscience, CA, USA) in DMEM/F12 medium supplemented with 10% FBS (v/v) and 1% (v/v) L-glutamine-penicillin-streptomycin (Life Technologies, CA, USA) until 80% confluence, then scraped, and counted using trypan blue (BioRad) and TC20 Automated Cell Counter (BioRad). Subsequently, for proliferation analysis, 2 × 10^4^ cells/well were seeded on 96-well plates (Corning CellBIND surface; Corning, NY, USA) and cultured until 80% confluence at 37°C in a humidified atmosphere containing 5% CO_2_, at which point medium was replaced with a fresh one with or without 10% FBS (two variants of the experiment) and 0, 20, 100, or 500 ng/mL of recombinant visfatin (PeproTech, NJ, USA) for 8 or 24 hours. For Nampt/PBEF/visfatin expression analysis, 3 × 10^5^ cells/well were seeded on plastic, flat bottom, 6-well plates (Corning CellBIND surface) and cultured until 80% confluence at 37°C in a humidified atmosphere containing 5% CO_2_. Next, depending on the nature of the experiment, the complete medium was replaced with fresh one: (a) devoid of serum, except for control cells (serum-supplemented), and grown for 12, 24, and 48 hours in serum withdrawal experiment; (b) devoid of serum in serum induction experiment, all cells being grown without serum for 24 h and subsequently medium being changed into serum-supplemented (10% FBS) in all cells except for controls, which were supplemented again with fresh serum-free medium, and harvested after 24 or 48 hours; (c) containing 0, 50, 100, or 200 *μ*M CoCl_2_ (Alchem, Poland) for 4, 8, 12, and 24 hours in hypoxia experiment. Upon termination, cells were scratched and lysed with 1 mL of TRIzol Reagent (Life Technologies) and stored at −80°C until RNA isolation, as described in earlier sections. For reverse transcriptase reaction, 500 ng of isolated RNA per reaction was used.

#### 2.3.2. Proliferation Assay

Cell proliferation was measured as total protein synthesis using sulforhodamine B (SRB), an anionic dye binding electrostatically to cellular proteins and subsequently fixed, solubilized, and measured spectrophotometrically at OD 510 nm. SRB assays were conducted in four technical replicates and the experiment was conducted three times.

### 2.4. Statistical Analysis

In mRNA analysis, QBasePLUS version 2.4 software (Biogazelle BE, Belgium) [13] was used to calculate efficiency-corrected relative quantities normalized to the expression of reference genes for all samples (NRQ). All statistical analyses on NRQ were performed using MedCalc Statistical Software version 12.7.7 (MedCalc Software bvba, Ostend, Belgium; https://www.medcalc.org/; 2013). Normality and homogeneity of variances were tested using D'Agostino-Pearson and Levene's tests. Data were log-transformed and analyzed using one-way ANOVA and *t*-test for independent samples with Welch correction or Kruskal-Wallis *H* test and Mann-Whitney *U* test. Paired samples were analyzed using *t*-test for dependent samples. Correlation and partial correlation analyses were conducted using Pearson test (*r*) and correlation with circulating cytokines/growth factors using Spearman test (*ρ*). Two-factorial ANOVA, analysis of covariance (ANCOVA), and multiple regression (stepwise method; variable entrance criteria: *p* < 0.05; variable removal criteria: *p* > 0.1) were used to coevaluate factors/variables. Frequency analysis was conducted using Fisher test. If not otherwise stated, NRQ data are presented as geometric means with 95% confidence intervals (CI). In protein analysis, data are presented as means with 95% CI and as medians with 95% CI in circulating Nampt/PBEF/visfatin analysis and analyzed using, respectively, *t*-test (for paired or unpaired observations, the latter with Welch correction for unequal variances, if required) or Mann-Whitney *U* test. For cell culture experiments, ΔΔCt method using efficiency-corrected Cq values was used to evaluate relative differences in target gene expression. All calculated probabilities were two-sided and *p* < 0.05 was considered statistically significant.

## 3. Results

### 3.1. Nampt/PBEF/Visfatin Expression in Colorectal Tissue

**Figure 1:**
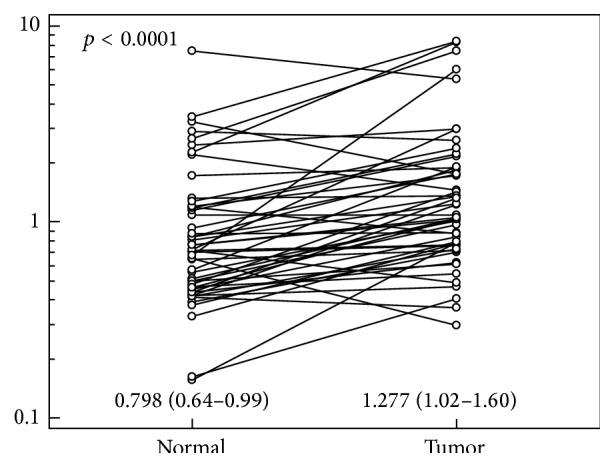
Pairwise comparison of Nampt/PBEF/visfatin expression between tumor and adjacent nonneoplastic (normal) colorectal tissue. Data presented as normalized relative quantities (NRQ: normalized against geometric mean of* GAPDH* and* PPIA* expression, referred to as mean expression across sample set investigated; calculated by qBasePLUS software) with 95% CI and analyzed using *t*-test for paired samples.

Normalized relative Nampt/PBEF/visfatin expression (NRQ) in tumors was significantly higher than in corresponding noncancerous tissue by 1.6-fold on average (95% CI: 1.4–1.9; maximum: 8.9-fold) (Figure 1). Tumor-to-normal Nampt/PBEF/visfatin expression ratio exceeded one in 78% of patients and in this group mean upregulation was 2-fold (1.7–2.3).

Nampt/PBEF/visfatin expression in tumors corresponded with disease advancement (Table 1). Compare to mean expression in nontumor tissue, Nampt/PBEF/visfatin expression in tumors was significantly higher in all disease stages except for stage I. It did not differ significantly with respect to local tumor progression but was higher in N2 than N1 cancers (2-fold upregulation (1.1–3.7), *p* = 0.029) and in M1 than M0 cancers (2.7-fold (1.3–5.3), *p* = 0.006). Similarly, Nampt/PBEF/visfatin expression in nonneoplastic tissue mirrored disease advancement. It was 2.1-fold upregulated (1.1–4.1, *p* = 0.033) in normal tissue in patients with N2 than N1 cancers and 2.2-fold upregulated (1.1–4.4, *p* = 0.026) in M1 than M0 cancers.

Nampt/PBEF/visfatin expression in both tumor and normal tissue was associated with anemia (Table 1). It was 1.6-fold (1–2.3, *p* = 0.033) upregulated in tumors from CRC patients with mild anemia compared to those without and 1.6-fold (1–2.5, *p* = 0.025) in normal tissue, while 2.7-fold (1–7, *p* = 0.046) downregulated in tumor tissue from patients with moderate/severe anemia as compared to nonanemic ones and 2.2-fold (1–4.7, *p* = 0.048) in normal tissue. However, colorectal expression of Nampt/PBEF/visfatin was strongly associated with* HIF1α* (Table 3) and* HIF1α* corresponded with anemia in normal tissue as well (*p* = 0.017). Hence, we used ANCOVA and found that for tumor tissue both* HIF1α* (*p* < 0.001) and anemia (*p* = 0.014) significantly affected Nampt/PBEF/visfatin expression. However, in nontumor tissue, the association with anemia lost significance (*p* = 0.718) when Nampt/PBEF/visfatin correlation with* HIF1α* (*p* < 0.001) was accounted for.

The highest mean Nampt/PBEF/visfatin expression was in tumors located in left colon and the fold-increase between normal and tumor tissue was the highest for this location (Table 1). Nampt/PBEF/visfatin expression in left colon tumors tended to be 1.7-fold higher (1–2.8, *p* = 0.055) as compared to tumors located in rectum. When the differences in TNM stage between left colonic and rectal cancers were accounted for (two-way ANOVA), higher Nampt/PBEF/visfatin expression in tumors located in left colon gained significance (*p* = 0.049). There was no Nampt/PBEF/visfatin upregulation in right colon tumors.

### 3.2. Nampt/PBEF/Visfatin Expression in Whole Blood

Nampt/PBEF/visfatin in whole blood was 1.3-fold (1–1.8) lower in CRC patients than in controls (0.840 (0.69–1.02) versus 1.123 (0.93–1.36), *p* = 0.043).

Relationship between Nampt/PBEF/visfatin in whole blood and clinicopathological features of CRC is depicted in Table 2. It tended to be 1.5-fold higher in CRC patients with N2 than N0 cancers (0.9–2.5, *p* = 0.114) or 1.9-fold higher in patients with distant metastases (0.9–3.8) and to be upregulated along with increasing depth of tumor invasion or in patients with anemia (1.4-fold (1–2.1), *p* = 0.069).

### 3.3. Correlation Pattern of Nampt/PBEF/Visfatin in Colorectal Tissue

We examined the association pattern between Nampt/PBEF/visfatin and colonic expression of proinflammatory cytokines* IL1β* and* TNFα*, chemokines* CCL2* and* CCL4*, and angiogenic factors* VEGF-A*,* FGF2*, and* IL8* and with indices of hypoxia,* HIF1α,* and proliferation,* PCNA*. Except for* PCNA* and* CCL4* in tumors, Nampt/PBEF/visfatin expression correlated positively with all, both in normal and tumor colonic tissue (Table 4). However, as revealed by partial correlation analysis, some of these associations were mediated by* HIF1α*. When adjusted, apart from* HIF1α*, Nampt/PBEF/visfatin in tumors correlated with* IL1β*,* VEGF-A*, and* IL8*. Following adjustment to* HIF1α*, Nampt/PBEF/visfatin in normal tissue correlated with* IL1β*,* IL8*,* CCL2*, and* CCL4*.

In multivariate analysis with* HIF1α, IL1β*,* VEGF-A*,* IL8*, mild anemia, and M status as explanatory variables,* HIF1α* (*b* = 0.61; partial *r* = 0.66, *p* < 0.001),* IL1β* (*b* = 0.23; partial *r* = 0.45, *p* = 0.001), and mild anemia (*b* = 0.11; partial *r* = 0.29, *p* = 0.043) were found to be independent predictors of tumor Nampt/PBEF/visfatin, explaining 70% in its variability (coefficient of determination *R* ^2^ = 0.703; *F* = 37.1, *p* < 0.0001; constant = −0.03).

In multivariate analysis with* HIF1α, IL1β*,* IL8*,* CCL2*,* CCL4,* and stage M as explanatory variables,* IL1β* (*b* = 0.39; partial *r* = 0.65, *p* < 0.001) and* HIF1α* (*b* = 0.44; partial *r* = 0.42, *p* = 0.002) were found to be independent predictors of Nampt/PBEF/visfatin in nonneoplastic tissue, explaining 73% in its variability (coefficient of determination *R* ^2^ = 0.725; *F* = 64.5, *p* < 0.0001; constant = 0.007).

In a subset of CRC patients (*n* = 44), we examined Nampt/PBEF/visfatin correlation with serum levels of IL1*β*, IL4, IL12, IL6, IL8, TNF*α*, CCL2, CCL4, VEGF-A, PDGF-BB, FGF2, G-CSF, and GM-CSF. Of these tumor Nampt/PBEF/visfatin positively correlated with circulating IL8 (*ρ* = 0.33, *p* = 0.029) and VEGF-A (*r* = 0.40, *p* = 0.008).

### 3.4. Correlation Pattern of Nampt/PBEF/Visfatin in Whole Blood

There was no correlation between whole blood Nampt/PBEF/visfatin and its expression in nonneoplastic (*r* = 0.04, *p* = 0.880, *n* = 20) or tumor tissue (*r* = 0.11, *p* = 0.644, *n* = 20).

Whole blood Nampt/PBEF/visfatin positively correlated with* IL1β* (*r* = 0.56, *p* < 0.001),* IL8* (*r* = 0.43, *p* = 0.008), and* CCL4* (*r* = 0.43, *p* = 0.010) but not with* TNFα* (*r* = −0.11, *p* = 0.535) or* VEGF-A* (*r* = 0.32, *p* = 0.100).

We also investigated Nampt/PBEF/visfatin correlation with biochemical and hematological parameters: CRP, ESR, fibrinogen, PLT, WBC, hemoglobin, hematocrit, and RBC. Of these, Nampt/PBEF/visfatin expression correlated with hemoglobin (*r* = −0.33, *p* = 0.049) and hematocrit (*r* = −0.36, *p* = 0.032).

Whole blood Nampt/PBEF/visfatin expression positively correlated exclusively with circulating IL6 (*r* = 0.37, *p* = 0.039, *n* = 32).

In multivariate analysis with* IL1β*,* IL8, CCL4,* hemoglobin, and hematocrit as explanatory variables,* IL1β* (*b* = 0.81; partial *r* = 0.65, *p* < 0.001),* IL8* (*b* = 0.28; partial *r* = 0.56, *p* < 0.001), and hematocrit (*b* = −0.02; partial *r* = −0.46, *p* = 0.007) were found independent predictors of Nampt/PBEF/visfatin expression in whole blood, explaining 60% in its variability (coefficient of determination *R* ^2^ = 0.5983; *F* = 15.4, *p* < 0.0001; constant = 0.649).

### 3.5. Nampt/PBEF/Visfatin Protein Concentration in Bowel Tissue

**Figure 2:**
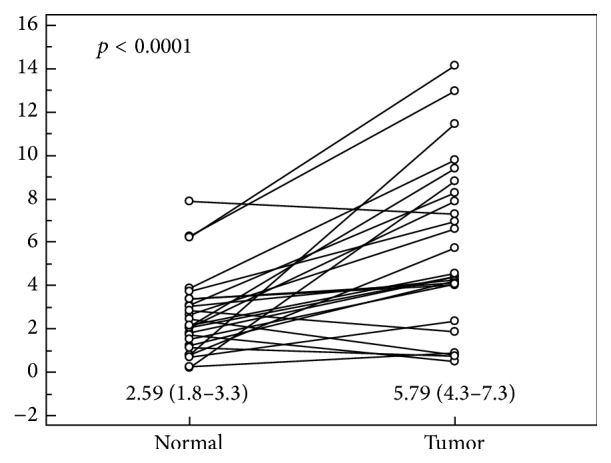
Pairwise comparison of Nampt/PBEF/visfatin protein concentration between tumor and adjacent nonneoplastic (normal) colorectal tissue. Data presented as relative Nampt/PBEF/visfatin concentrations, adjusted to total protein content, expressed in (mg/g), and analyzed using *t*-test for paired samples.

Relative Nampt/PBEF/visfatin protein concentration in tumors was higher by 2.4-fold (1.7–3) on average than in nontumor tissues (Figure 2). Nampt/PBEF/visfatin protein concentration in tumor tissue corresponded with CRC advancement; it was significantly higher in stage III/IV than I/II CRCs (7.8 mg/g (5–10.5) versus 4.5 mg/g (2.8–6.3), *p* = 0.030). Elevated concentration reflected metastatic spread, being higher in N1/2 than N1 CRCs (8.2 mg/g (4.7–11.6) versus 4.7 mg/g (3.1–6.3), *p* = 0.028), rather than local advancement (*p* = 0.4). Also Nampt/PBEF/visfatin protein concentration in tumor-adjacent normal tissue corresponded with the disease advancement. It tended to be higher in stage III/IV than I/II CRCs (3.6 mg/g (1.9–5.3) versus 2 mg/g (1.3–2.6), *p* = 0.064) and was significantly higher in N1/2 than N1 CRCs (4 mg/g (1.8–6.1) versus 2 mg/g (1.4–2.5), *p* = 0.009).

There was no direct correlation between Nampt/PBEF/visfatin protein concentration and its mRNA expression in terms of NRQ, neither in tumor nor in normal tissue. Also, its tissue protein concentration did not correlate with circulating cytokines and growth factors. However, in noncancerous tissues sampled from areas adjacent to resection margins, Nampt/PBEF/visfatin protein concentration was significantly higher in patients with anemia (3.04 mg/g (2.09–4) versus 1.37 mg/g (0.84–1.9), *p* = 0.003) and tended to negatively correlate with hemoglobin concentration (*r* = −0.37, *p* = 0.065).

### 3.6. Circulating (Serum) Nampt/PBEF/Visfatin

Circulating Nampt/PBEF/visfatin in CRC patients was significantly higher than in healthy controls (12.6 *μ*g/L (7.2–20.5) versus 6.7 *μ*g/L (4.8–9.3), *p* = 0.019). It tended to be more elevated in stage III/IV than stage I/II CRCs (22.9 *μ*g/L (5.8–64.60) versus 11 *μ*g/L (5.5–17.2), *p* = 0.133) and in T3/4 than T2 CRCs (17.6 *μ*g/L (8.2–23) versus 7.7 *μ*g/L (4.3–21.9), *p* = 0.222). However, circulating Nampt/PBEF/visfatin was significantly elevated in N1/2 than N0 CRCs (23.9 *μ*g/L (8.6–100) versus 9.2 *μ*g/L (5.2–15.9), *p* = 0.012).

Circulating Nampt/PBEF/visfatin positively correlated with inflammatory indices IL1*β* (*r* = 0.63, *p* = 0.020) and TNF*α* (*r* = 0.62, *p* = 0.024) and growth factors FGF2 (*r* = 0.74, *p* = 0.004), G-CSF (*r* = 0.62, *p* = 0.025), and GM-CSF (*r* = 0.57, *p* = 0.043). There was no direct correlation between circulating Nampt/PBEF/visfatin and its mRNA expression or protein concentration, either in tumor or in normal tissue.

### 3.7. Effect of Serum Deprivation and Induction as well as Hypoxia on Nampt/PBEF/Visfatin Expression in Colon Adenocarcinoma Cell Line SW620

The analysis was preceded by selecting optimal housekeeping genes using RefFinder algorithm (the website was accessed in 2014: http://www.leonxie.com/referencegene.php), based on the rankings from four dedicated computational programs, namely, geNorm, Normfinder, BestKeeper, and the comparative ΔCt method, as well as by determining required number of normalizers with GeNorm utility in qBasePLUS program [13] out of the following genes:* ACTB*,* B2M*,* GAPDH*,* GUSB*,* HPRT1*,* IPO8*,* MRPL19*,* PGK1*,* PPIA*,* RPLP0*,* RPS23*,* SDHA*,* TBP*,* UBC*, and* YWHAZ*.* YWHAZ* and* PPIA* were selected as optimal for SW620 cells in serum withdrawal experiment,* YWHAZ* and* IPO8* in serum induction experiment, and* SDHA* and* TBP* in hypoxia experiment.

**Figure 3:**
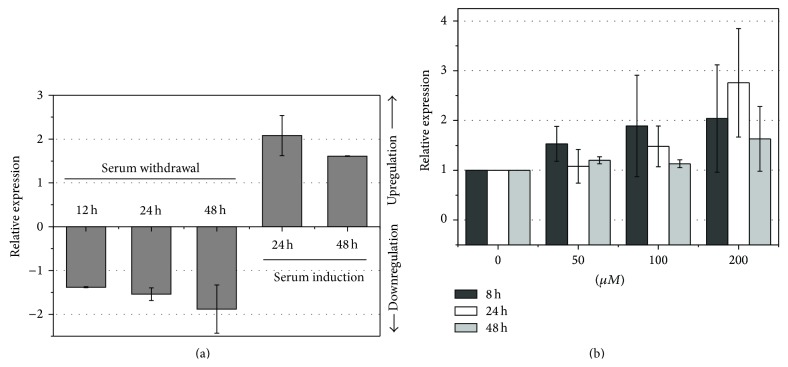
Effect of serum deprivation and induction as well as hypoxia on* Nampt/PBEF/visfatin expression* in colon adenocarcinoma cell line SW620. (a)* Effect of serum withdrawal and serum induction*. In a serum withdrawal experiment, cells were grown with 10% FBS to 80% confluence, at which point serum supplementation was withdrawn for 12, 24, and 48 hours; data are presented as relative expression of* Nampt* (mean ± SD of three independent biological experiments) in starved versus FBS-supplemented cells. In a serum induction experiment, cells were grown with 10% FBS to 80% confluence at which point serum supplementation was withdrawn and resupplemented after 24 hours for 24 or 48 hours in all cultures except for controls; data are presented as relative expression of* Nampt* (mean ± SD of independent biological experiments) in resupplemented versus FBS-starved cells. (b)* Effect of hypoxia*. Cells were grown for 80% confluence and stimulated with 50, 100, and 200 *μ*M concentrations of cobalt chloride, a hypoxia-mimetic agent, for 8, 24, and 48 hours. Data are presented as relative expression of* Nampt* (mean ± SD of three independent biological experiments) in stimulated compared to unstimulated cells.

We examined both the effect of serum withdrawal and serum resupplementation on SW620 cells. Compared to cells supplemented with 10% FBS, serum withdrawal was associated with reduced expression of* Nampt/PBEF/visfatin*, in a time-dependent manner. In turn, serum induction resulted in an upregulation of* Nampt/PBEF/visfatin* expression in starved SW620 cells (Figure 3(a)). Similarly, cytokine expression was enhanced in SW620 cells grown under hypoxic conditions, particularly in 24-hour cultures stimulated with 200 *μ*M CoCl_2_ (Figure 3(b)).

### 3.8. Effect of Exogenous Visfatin on Growth of Adenocarcinoma Cell Line SW620

**Figure 4:**
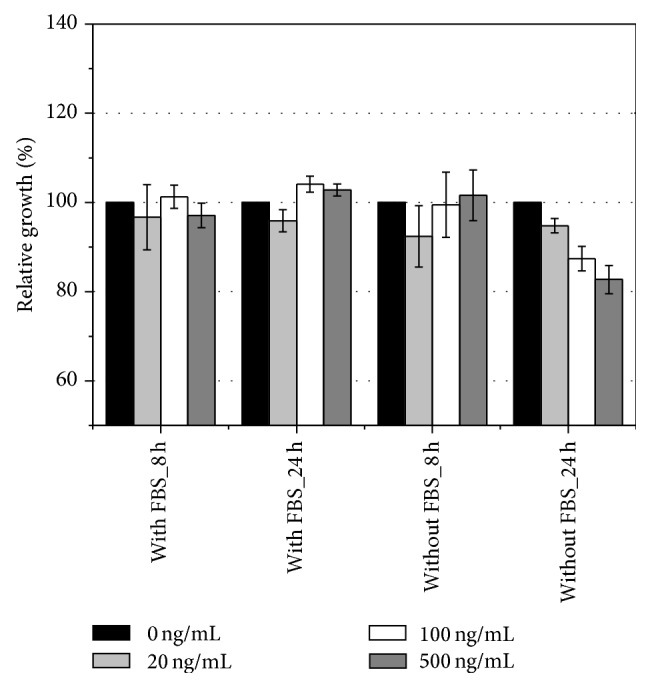
Effect of exogenous visfatin on growth of colon adenocarcinoma cell line SW620. Cells were grown with 10% FBS to 80% confluence and subsequently medium had been changed on fresh serum-supplemented or serum-free medium with 0, 20, 100, and 500 ng/mL recombinant visfatin, in which cells were grown for 8 or 24 hours. Results are presented as relative growth of stimulated versus unstimulated (100%) cells, determined with SRB assay.

As depicted in Figure 4, stimulation with recombinant visfatin did not provide growth advantage for adenocarcinoma cell line SW620. On the contrary, 24-hour stimulation with visfatin caused, dose-dependently, growth inhibition of SW620 cells grown without serum supplementation.

## 4. Discussion

To the best of our knowledge this is the first study dedicated to Nampt/PBEF/visfatin expression in colorectal tissue and whole blood from CRC patients and its association with the disease advancement, identifying factors possibly involved in its upregulation. The link between colorectal tissue Nampt/PBEF/visfatin and CRC has been discovered by Hufton and colleagues [5, 6]. Nampt/PBEF/visfatin association with CRC advancement has been demonstrated for extracellular Nampt in some [7] but not all studies [8, 9]. We showed significant Nampt/PBEF/visfatin upregulation in tumor tissue and an increase of Nampt/PBEF/visfatin expression along with CRC advancement. However, its upregulation was associated with gaining metastatic potential rather than with local progression, being higher in N2 than N1 and in M1 than M0 cancers. Higher than expected expression in N0 cancers, reflected also in higher expression in disease stage II than III, may result from failing to detect metastatic lymph nodes or presence of micrometastases and hence misclassification of some cancers. Our observations were confirmed on protein level as well and the association between Nampt/PBEF/visfatin and lymph node involvement in particular was further supported by significant elevation of circulating cytokine in N-positive CRCs. Possible role of Nampt/PBEF/visfatin in metastasis has been suggested for prostate cancer, based on an observation of Nampt/PBEF/visfatin-mediated upregulation of expression and activity of matrix metalloproteinases- (MMP-) 2 and 9 [14].

Tissue adjacent to resection margins, which were confirmed to be disease-free by histopathological examination, was affected by disease progression as well. This unexpected observation might be explained by a phenomenon of field effect in cancer. Although generally higher in tumors, even stage II or T2, Nampt/PBEF/visfatin expression in this apparently normal colorectal tissue was not uniformly low. It differed by the disease stage and reflected cancer metastatic potential, mimicking the associations observed in tumors. Similar to tumors, Nampt/PBEF/visfatin expression in nontumor tissues was more pronounced in N-positive cancers also on protein level. A phenomenon of field cancerization is well documented now, also in CRC [15], and a number of genetic and transcriptional alterations in histologically normal tissue adjacent to tumor that mirror these observed in cancer have been found.

Owing to a very high rate of NAD^+^ turnover in cancer cells, Nampt/PBEF/visfatin is viewed as a potential target of future anticancer therapies and several potent enzyme inhibitors have already been developed [3]. While the preclinical studies with the compounds yielded promising results and Nampt/PBEF/visfatin inhibitors have entered clinical trials as anticancer agents [3], surprisingly little is known about physiological role or relevance of Nampt/PBEF/visfatin or mechanisms underlying its actions. In gastrointestinal tract cancers, targeting enzymatic activity of Nampt/PBEF/visfatin has inhibited proliferation, migration, and anchorage-independent growth [16, 17] and induced apoptosis [16–19], implying Nampt/PBEF/visfatin involvement in proliferation, migration, and survival of cancer cells. To address the issue in actual tumors, we investigated Nampt/PBEF/visfatin association with the expression of PCNA, a surrogate proliferation marker. However, in line with poor correlation between Nampt/PBEF/visfatin and T stage, Nampt/PBEF/visfatin did not correspond with* PCNA* expression. Furthermore, stimulation with exogenous visfatin not only did not provide growth advantage for colon adenocarcinoma cell line SW620, but, following 24-hour incubation, hampered it in a dose-dependent manner.

Peripheral blood leukocytes have been reported to contain maximal levels of mRNA transcripts for Nampt/PBEF/visfatin and greatly contribute to its circulating levels [10]. Nampt/PBEF/visfatin is essential for survival of both activated [20–22] and resting leukocytes [4]. We found that whole blood expression of Nampt/PBEF/visfatin in CRC patients tended to echo advancement of the disease but, surprisingly, we found it to be lower than in controls. Blood samples for the control group were collected from patients with nonmalignant diseases of gastrointestinal tract. While these patients were proved to be reliable reference for studies on circulating cytokines, it is possible that their whole blood cytokine expression is altered. Contrary to its whole blood expression, circulating visfatin was significantly higher in CRC patients than controls, reflecting lymph node involvement.

Nampt/PBEF/visfatin is both stimulated by inflammatory stimuli and increases expression of inflammatory cytokines in return [10, 23]. Correspondingly, we found Nampt/PBEF/visfatin to positively correlate with the expression of inflammatory mediators with* IL1β* being an independent predictor for its tumor and normal colorectal tissue expression and* IL1β* and* IL8* for its expression in whole blood. In line with its proposed function as a chemoattractant for leukocytes [24], we observed Nampt/PBEF/visfatin expression to correlate with other chemokines, namely, monocyte chemotactic protein- (MCP-) 1 (CCL2) and macrophage inflammatory protein- (MIP-) 1*β* (CCL4). Moreover, we found circulating IL8 and IL6 to correspond with, respectively, tumor and whole blood expression of Nampt/PBEF/visfatin. Also the levels of circulating cytokine were tightly related to these of IL*β* and TNF*α*.

*In vitro* studies have shown that Nampt/PBEF/visfatin dose-dependently upregulated HUVECs' VEGF-A expression, contributing to endothelial angiogenesis [25]. Accordingly, we demonstrated Nampt/PBEF/visfatin to positively correlate with circulating VEGF-A as well as with* VEGF-A* expression in tumor and normal colorectal tissue. Corroborating finding on its positive effect on FGF-2 mRNA levels in human endothelial cells [26], we found Nampt/PBEF/visfatin to positively correlate with FGF2 as well. Also at systemic level, there was strong positive correlation between cytokine and FGF2 as well as G-CSF and GM-CSF, hematopoietic growth factors with angiogenic properties.

Hypoxia is a common phenomenon within solid tumors and a driving force of tumor angiogenesis with hypoxia-inducible factor- (HIF-) 1 as a key regulatory factor of the process. Nampt/PBEF/visfatin has been proved to be yet another hypoxia-inducible factor with its expression regulated by HIF-1 [27, 28]. Accordingly, we demonstrated a strong positive association between Nampt/PBEF/visfatin and* HIF1α* expression, both in tumor and normal tissue, with* HIF1α* being one of independent predictors of its expression in CRC. Also,* HIF1α* occurred to be a confounding factor mediating some of other Nampt/PBEF/visfatin associations observed in our study. It did not, however, mediate the association between tumor Nampt/PBEF/visfatin and anemia, although systemic hypoxia with subsequent upregulation of* HIF1α* would be the simplest explanation for an increase in Nampt/PBEF/visfatin. Not only was Nampt/PBEF/visfatin expression higher in tumors in CRC patients with mild anemia but also whole blood Nampt/PBEF/visfatin inversely correlated with hemoglobin and hematocrit, with the latter being independently associated with Nampt/PBEF/visfatin. Interestingly, at protein level, anemia was associated with higher Nampt/PBEF/visfatin concentration in noncancerous bowel tissue as well. Anemia of chronic disease is accompanied by elevation of inflammatory mediators that by various, although still not fully clarified, mechanisms may contribute to its development. However, the association observed here seems to be independent from inflammation as well since IL1*β* was Nampt/PBEF/visfatin independent predictor in addition to anemia/hematocrit. Owing to the importance of Nampt/PBEF/visfatin in cell bioenergetics, intuitively, its underexpression is likely to contribute to anemia. Accordingly, fatigue and anemia are listed among side-effects of treatment with Nampt inhibitors [29]. Yet, in the logistic regression, of all the factors investigated,* Nampt/PBEF/visfatin* upregulation in addition to* HIF1α* was an anemia predictor in CRC patients (data not shown). Our* in vitro* experiments confirmed that hypoxic conditions contribute to the upregulation of* Nampt/PBEF/visfatin* expression. Moreover, we showed that its expression is affected by the alterations in serum availability as well. Faulty blood vessels in tumors are not only inefficient in providing cancer cells with oxygen but are also responsible for discontinuity of supply of nutrients and growth factors. As such, cancer cells are likely to compensate by altering their expression profile. Indeed, both serum withdrawal and subsequent induction has been demonstrated to alter the expression of a number of genes [30]. The expression of* GAPDH* or* ACTB*, genes previously believed to be unregulated and as such frequently used as normalizers in gene expression studies, has been affected by serum availability as well. We found Nampt/PBEF/visfatin expression to be negatively affected by serum withdrawal in SW620 cells but upregulated in response to serum induction.

Specific location within colorectum has been shown to determine expression of a number of cytokines and growth factors, the phenomenon that may explain site differences in mechanisms promoting CRC progression, its recurrence pattern, patients' prognosis, or effectiveness of various treatment strategies [31, 32]. Indeed, there was no upregulation of Nampt/PBEF/visfatin in tumors located in right colon while there was over 2-fold upregulation in tumors located in left colon. Also, we observed significantly higher Nampt/PBEF/visfatin overexpression in tumors located in left colon than in rectum.

Van Beijnum et al. [6] has demonstrated that Nampt/PBEF/visfatin expression evaluated on transcriptional and protein level, the latter using either Western-blot or immunochemistry, was concordant. Accordingly, we found the magnitude of its upregulation on mRNA and protein level, both determined using sensitive quantitative methods, namely, RT-qPCR and flow cytometry-based Luminex technology, comparable. Although there was no direct correlation between cytokine expression on mRNA and protein level, cancer dissemination was associated with the upregulation of both Nampt/PBEF/visfatin protein and mRNA. Moreover, with circulating visfatin significantly higher in patients with lymph node involvement, the association manifested itself at systemic level as well. Also the association with anemia and inflammatory mediators, discerned on transcriptional level both in bowel and in peripheral leukocytes, has been confirmed on systemic, protein level.

## 5. Conclusions

Taken together our results show Nampt/PBEF/visfatin overexpression in colorectal cancer that, both on transcriptional and protein level, locally and at systemic level, reflects metastatic potential of the disease and points at inflammatory stimuli and hypoxia as key inducers of its upregulation in CRC. They also demonstrate that the disease stage is reflected by Nampt/PBEF/visfatin upregulation in nontumor apparently normal colorectal tissue.

## Figures and Tables

**Table 1:** Relationship between Nampt/PBEF/visfatin expression in colorectal tissue and clinicopathological features of CRC.

Parameter	*N*	Nampt/PBEF/visfatin		Ratio T/N
		Tumor tissue	Nontumor tissue	
Sex distribution, F/M ratio		17/35		
Age, median (range)		68 yrs (33–91)		
Disease stage:				
I	10	0.973 (0.74–1.27)^e^	0.475 (0.32–0.72)^c,d,e^	2.1 (1.4–3.1)^f^
II	20	1.270 (0.83–1.95)^a^	0.903 (0.63–1.29)^b,d,e^	1.4 (1-2)^f^
III	16	1.133 (0.85–1.52)^a,e^	0.743 (0.52–1.06)^b,c,e^	1.5 (1.3–1.9)^f^
IV	5	3.074 (0.95–9.90)^a,b,d^	1.615 (0.65–3.99)^b,c,d^	1.9 (0.8–4.6)
Local advancement (T):				
T2	13	1.217 (0.88–1.69)^a^	0.565 (0.37–0.85)	2.2 (1.6–3.1)^f^
T3	21	1.226 (0.83–1.82)^a^	0.874 (0.61–1.25)	1.4 (1–1.9)^f^
T4	17	1.365 (0.88–2.11)^a^	0.901 (0.62–1.30)	1.5 (1.2–1.9)^f^
Lymph node metastases (N):				
N0	33	1.259 (0.94–1.69)	0.775 (0.59–1.02)	1.6 (1.3–2.1)^f^
N1	11	0.983 (0.69–1.41)^g^	0.627 (0.40–0.98)^g^	1.6 (1.3–2.1)^f^
N2	7	1.963 (1.04–3.72)^h^	1.308 (0.76–2.25)^h^	1.5 (1–2.3)^f^
Distant metastases (M):				
M0	46	1.152 (0.93–1.42)^i^	0.734 (0.59–0.91)^i^	1.6 (1.3–1.9)^f^
M1	5	3.074 (0.95–9.90)^j^	1.615 (0.65–3.99)^j^	1.9 (0.8–4.6)
Anemia:				
Nonanemic	20	1.050 (0.84–1.31)^k,l^	0.639 (0.48–0.85)^k,l^	1.6 (1.3–2.1)^f^
Mild anemia	26	1.637 (1.15–2.33)^l,m^	1.039 (0.76–1.43)^l,m^	1.6 (1.2–2)^f^
Moderate/severe	5	0.716 (0.26–2.01)^k,m^	0.475 (0.20–1.15)^k,m^	1.5 (0.6–3.9)
Tumor location:				
Left	15	1.778 (1.14–2.77)	0.772 (0.46–1.28)	2.2 (1.5–3.3)^f^
Right	18	1.135 (0.76–1.70)	0.945 (0.66–1.35)	1.2 (0.9–1.6)
Rectum	18	1.070 (0.77–1.49)	0.666 (0.51–0.88)	1.6 (1.4–1.9)^f^

Data presented as means with 95% CI of normalized (against geometric mean of *GAPDH* and *PPIA *expression) relative (referred to as mean expression across sample set investigated) quantities of Nampt/PBEF/visfatin, calculated by qBasePLUS software. Information in superscript indicates significant (*p* < 0.05) between-group differences calculated using one-way ANOVA and *t*-test for independent samples. Ratio of tumor-to-normal (T/N) expression for each parameter was calculated using *t*-test for paired samples. Data presented as mean upregulation with 95% confidence interval.

^a^Different from normal tissue; ^b^different from stage I; ^c^different from stage II; ^d^different from stage III, ^e^different from stage IV; ^f^significantly upregulated in tumor tissues compared to matched nontumor ones (*t*-test for paired samples); ^g^different from N2 cancers; ^h^different from N1 cancers; ^i^different from M1 cancers; ^j^different from M0 cancers; ^k^different from mild anemia; ^l^different from moderate/severe anemia; ^m^different from nonanemic patients.

**Table 2:** Relationship between Nampt/PBEF/visfatin expression in whole blood and clinicopathological features of CRC.

Parameter	*N*	Nampt/PBEF/visfatin	*p* value
Sex distribution, F/M ratio	36	CRC patients: 8/28	0.367
	54	Controls: 18/36	
Age, median (range)		CRC patients: 62 (33–92)	<0.001
		Controls: 36.5 (18–78)	
Disease stage:			0.126
I	3	0.482 (0.11–2.06)	
II	11	0.824 (0.54–1.26)	
III	18	0.852 (0.65–1.12)	
IV	3	1.505 (0.75–3.06)	
Not determined	1	1.108	
Local advancement (T):			0.090
T2	4	0.495 (0.23–1.06)	
T3	13	0.809 (0.55–1.19)	
T4	19	0.984 (0.77–1.25)	
Lymph node metastases (N):			0.232
N0	15	0.713 (0.52–0.98)	
N1	13	0.915 (0.64–1.31)	
N2	8	1.076 (0.69–1.67)	
Distant metastases (M):			0.073
M0	32	0.798 (0.65–0.98)	
M1	3	1.505 (0.75–3.03)	
M*x*	1	1.108	
Anemia:			0.069
Nonanemic	21	0.732 (0.56–0.96)	
Mild/moderate anemia	15	1.045 (0.80–1.37)	

Data presented as means with 95% CI of normalized (against geometric mean of *SDHA* and *TBP *expression) relative (referred to as mean expression across sample set investigated) quantities of Nampt/PBEF/visfatin, calculated by qBasePLUS software.

**Table 3:** Sequences and efficiencies of primers used in current study.

Symbol	Gene name	Accession number	Primer sequence 5′→3′ (forward/reverse)	Amp. size	*E* [%] tissue	*E* [%] blood	*E* [%] cells
*GAPDH* ^a^	Glyceraldehyde-3-phosphate dehydrogenase	NM_002046.4	F: gtctcctctgacttcaacagcg R: accaccctgttgctgtagccaa	131 bp	102.1	—	—

*IPO8 *	Importin 8; nuclear protein import	NM_006390.3	F: tggtatggtggaagtgtaagaagtg R: ttggttgagatagttgaatgcttgc	230 bp	—	—	100.9

*PPIA* ^a^	Peptidylprolyl isomerase A	NM_021130.3	F: ggcaaatgctggacccaacaca R: tgctggtcttgccattcctgga	161 bp	99.7	—	104.6

*SDHA *	Succinate dehydrogenase subunit A	NM_004168.2	F: agaggcacggaaggagtcac R: caccacatcttgtctcatcagtagg	267 bp	—	94.8	95.9

*TBP *	TATA-box-binding protein	NM_003194.4	F: tataatcccaagcggtttgctg R: ctggctcataactactaaattgttg	283 bp	—	109.7	102.2

*YWHAZ *	Tyrosine 3-monooxygenase/tryptophan 5-monooxygenase activation protein, zeta polypeptide; signal transduction	NM_003406.3	F: tcacaacaagcataccaagaagc R: gtatccgatgtccacaatgtcaag	263 bp	—	—	97.4

*NAMPT *	Nicotinamide phosphoribosyltransferase	NM_005746.2	F: cacaggcaccactaataatcagac R: ctccaccagaaccgaaggc	243 bp	104	108.8	94.8

*IL1β* ^a^	Interleukin 1*β*	NM_000576.2	F: ccacagaccttccaggagaatg R: gtgcagttcagtgatcgtacagg	131 bp	94.7	100.1	—

*IL8 *	Interleukin 8	NM_000584.3	F: caacacagaaattattgtaaagc R: aagtgttgaagtagatttgc	191 bp	96.7	99.8	—

*CCL2 *	Monocyte chemotactic protein- (MCP-) 1	NM_002982.3	F: tctgtgcctgctgctcatag R: acttgctgctggtgattcttc	155 bp	99.7	—	—

*CCL4* ^a^	Macrophage inflammatory protein- (MIP-) 1*β*	NM_002984.2	F: ggtcatacacgtactcctggac R: gcttcctcgcaactttgtggtag	140 bp	92.1	103.5	—

*FGF2 *	Basic fibroblast growth factor	NM_002006.4	F: tctatcaaaggagtgtgtgctaacc R: tgcccagttcgtttcagtgc	179 bp	100.7	—	—

*HIF1α*	Hypoxia-inducible factor 1*α*	NM_001530.3	F: ctgccaccactgatgaatta R: gtatgtgggtaggagatgga	90 bp	104.7	—	—

*PCNA* ^a^	Proliferating cell nuclear antigen	NM_002592.2	F: caagtaatgtcgataaagaggagg R: gtgtcaccgttgaagagagtgg	126 bp	100.8	—	—

*TNFα* ^a^	Tumor necrosis factor *α*	NM_000594.3	F: ctcttctgcctgctgcactttg R: atgggctacaggcttgtcactc	135 bp	98.2	100.1	—

*VEGF-A* ^a^	Vascular endothelial growth factor A	NM_001025366.2	F: ttgccttgctgctctacctcca R: gatggcagtagctgcgctgata	126 bp	96.1	94.5	—

Amp., amplicon; *E*, efficiency; ^a^primer sequences were as proposed by Origene (http://www.origene.com/). Remaining primers were designed using Beacon Designer Probe/Primer Design Software (BioRad), validated in silico by Blast analysis, and their specificity was tested by means of melting curve analysis and an electrophoresis in a high-resolution agarose (SeaKem LE agarose, Lonza, Switzerland) in TBE with SYBR Green (Lonza) detection. Efficiencies were calculated on pooled cDNA, separately for expression analysis in whole blood, colorectal tissue, and cell culture experiments.

Forward and reverse primer sequences are denoted by “F” and “R,” respectively.

**Table 4:** Correlation pattern of Nampt/PBEF/visfatin in normal and tumor tissue from CRC patients.

Gene	Normal tissue	Tumor tissue
*IL1β*	*r* = 0.82, *p* < 0.001^a^ (0.65, *p* < 0.001)	*r* = 0.65, *p* < 0.001^a^ (0.46, *p* < 0.001)
*TNFα*	*r* = 0.50, *p* < 0.001	*r* = 0.54, *p* < 0.001
*HIF1α*	*r* = 0.73, *p* < 0.001	*r* = 0.77, *p* < 0.001
*VEGF-A *	*r* = 0.66, *p* < 0.001	*r* = 0.64, *p* < 0.001^a^ (0.37, *p* = 0.008)
*PCNA *	*r* = 0.18, *p* = 0.200	*r* = −0.04, *p* = 0.774
*CCL4 (MIP1β) *	*r* = 0.62, *p* < 0.001^a^ (0.50, *p* < 0.001)	*r* = 0.24, *p* = 0.097
*CCL2 (MCP1) *	*r* = 0.70, *p* < 0.001^a^ (0.56, *p* < 0.001)	*r* = 0.57, *p* < 0.001
*IL8 *	*r* = 0.64, *p* < 0.001^a^ (0.39, *p* = 0.005)	*r* = 0.49, *p* < 0.001^a^ (0.38, *p* = 0.007)
*FGF2 *	*r* = 0.40, *p* = 0.004	*r* = 0.50, *p* < 0.001

^a^Associations that remained significant following adjustment to *HIF1α* with partial correlation coefficients given in parentheses.

## References

[1] Imai S.-I. (2009). Nicotinamide phosphoribosyltransferase (Nampt): a link between NAD biology, metabolism, and diseases. *Current Pharmaceutical Design*.

[2] Bi T.-Q., Che X.-M. (2010). Nampt/PBEF/visfatin and cancer. *Cancer Biology & Therapy*.

[3] Galli U., Travelli C., Massarotti A. (2013). Medicinal chemistry of nicotinamide phosphoribosyltransferase (NAMPT) inhibitors. *Journal of Medicinal Chemistry*.

[4] Pittelli M., Cavone L., Lapucci A. (2014). Nicotinamide phosphoribosyltransferase (NAMPT) activity is essential for survival of resting lymphocytes. *Immunology and Cell Biology*.

[5] Hufton S. E., Moerkerk P. T., Brandwijk R., De Bruïne A. P., Arends J.-W., Hoogenboom H. R. (1999). A profile of differentially expressed genes in primary colorectal cancer using suppression subtractive hybridization. *FEBS Letters*.

[6] Van Beijnum J. R., Moerkerk P. T. M., Gerbers A. J. (2002). Target validation for genomics using peptide-specific phage antibodies: a study of five gene products overexpressed in colorectal cancer. *International Journal of Cancer*.

[7] Nakajima T. E., Yamada Y., Hamano T. (2010). Adipocytokines as new promising markers of colorectal tumors: adiponectin for colorectal adenoma, and resistin and visfatin for colorectal cancer. *Cancer Science*.

[8] Fazeli M. S., Dashti H., Akbarzadeh S. (2013). Circulating levels of novel adipocytokines in patients with colorectal cancer. *Cytokine*.

[9] Chen M., Wang Y., Li Y. (2013). Association of plasma visfatin with risk of colorectal cancer: an observational study of Chinese patients. *Asia-Pacific Journal of Clinical Oncology*.

[10] Luk T., Malam Z., Marshall J. C. (2008). Pre-B cell colony-enhancing factor (PBEF)/visfatin: a novel mediator of innate immunity. *Journal of Leukocyte Biology*.

[11] Krzystek-Korpacka M., Diakowska D., Bania J., Gamian A. (2014). Expression stability of common housekeeping genes is differently affected by bowel inflammation and cancer: implications for finding suitable normalizers for inflammatory bowel disease studies. *Inflammatory Bowel Diseases*.

[12] Krzystek-Korpacka M., Diakowska D., Kapturkiewicz B., Bebenek M., Gamian A. (2013). Profiles of circulating inflammatory cytokines in colorectal cancer (CRC), high cancer risk conditions, and health are distinct. Possible implications for CRC screening and surveillance. *Cancer Letters*.

[13] Vandesompele J., de Preter K., Pattyn F. (2002). Accurate normalization of real-time quantitative RT-PCR data by geometric averaging of multiple internal control genes. *Genome Biology*.

[14] Patel S. T., Mistry T., Brown J. E. P. (2010). A novel role for the adipokine visfatin/pre-B cell colony-enhancing factor 1 in prostate carcinogenesis. *Peptides*.

[15] Hawthorn L., Lan L., Mojica W. (2014). Evidence for field effect cancerization in colorectal cancer. *Genomics*.

[16] Chini C. C. S., Guerrico A. M. G., Nin V. (2014). Targeting of NAD metabolism in pancreatic cancer cells: potential novel therapy for pancreatic tumors. *Clinical Cancer Research*.

[17] Bi T.-Q., Che X.-M., Liao X.-H. (2011). Overexpression of Nampt in gastric cancer and chemopotentiating effects of the Nampt inhibitor FK866 in combination with fluorouracil. *Oncology Reports*.

[18] Cerna D., Li H., Flaherty S., Takebe N., Coleman C. N., Yoo S. S. (2012). Inhibition of nicotinamide phosphoribosyltransferase (NAMPT) activity by small molecule GMX1778 regulates reactive oxygen species (ROS)-mediated cytotoxicity in a p53- and nicotinic acid phosphoribosyltransferase1 (NAPRT1)-dependent manner. *The Journal of Biological Chemistry*.

[19] Zhang C., Tong J., Huang G. (2013). Nicotinamide phosphoribosyl transferase (Nampt) is a target of microRNA-26b in colorectal cancer cells. *PLoS ONE*.

[20] Jia S. H., Li Y., Parodo J. (2004). Pre-B cell colony-enhancing factor inhibits neutrophil apoptosis in experimental inflammation and clinical sepsis. *Journal of Clinical Investigation*.

[21] Li Y., Zhang Y., Dorweiler B. (2008). Extracellular Nampt promotes macrophages survival via a non-enzymatic interleukin-6/ STAT3 signaling mechanism. *The Journal of Biological Chemistry*.

[22] Rongvaux A., Galli M., Denanglaire S. (2008). Nicotinamide phosphoribosyl transferase/pre-B cell colony-enhancing factor/visfatin is required for lymphocyte development and cellular resistance to genotoxic stress. *Journal of Immunology*.

[23] Neubauer K., Krzystek-Korpacka M. (2010). Visfatin/PBEF/Nampt and other adipocytokines in inflammatory bowel disease. *Advances in Clinical and Experimental Medicine*.

[24] Moschen A. R., Kaser A., Enrich B. (2007). Visfatin, an adipocytokine with proinflammatory and immunomodulating properties. *The Journal of Immunology*.

[25] Adya R., Tan B. K., Punn A., Chen J., Randeva H. S. (2008). Visfatin induces human endothelial VEGF and MMP-2/9 production via MAPK and PI3K/Akt signalling pathways: novel insights into visfatin-induced angiogenesis. *Cardiovascular Research*.

[26] Bae Y.-H., Bae M.-K., Kim S.-R., Lee J. H., Wee H.-J., Bae S.-K. (2009). Upregulation of fibroblast growth factor-2 by visfatin that promotes endothelial angiogenesis. *Biochemical and Biophysical Research Communications*.

[27] Bae S.-K., Kim S.-R., Kim J. G. (2006). Hypoxic induction of human visfatin gene is directly mediated by hypoxia-inducible factor-1. *FEBS Letters*.

[28] Segawa K., Fukuhara A., Hosogai N. (2006). Visfatin in adipocytes is upregulated by hypoxia through HIF1*α*-dependent mechanism. *Biochemical and Biophysical Research Communications*.

[29] Holen K., Saltz L. B., Hollywood E., Burk K., Hanauske A.-R. (2008). The pharmacokinetics, toxicities, and biologic effects of FK866, a nicotinamide adenine dinucleotide biosynthesis inhibitor. *Investigational New Drugs*.

[30] Pirkmajer S., Chibalin A. V. (2011). Serum starvation: caveat emptor. *The American Journal of Physiology—Cell Physiology*.

[31] Minoo P., Zlobec I., Peterson M., Terracciano L., Lugli A. (2010). Characterization of rectal, proximal and distal colon cancers based on clinicopathological, molecular and protein profiles. *International Journal of Oncology*.

[32] Krzystek-Korpacka M., Diakowska D., Grabowski K., Gamian A. (2012). Tumor location determines midkine level and its association with the disease progression in colorectal cancer patients: a pilot study. *International Journal of Colorectal Disease*.

